# Challenges in the Diagnosis of Magnesium Status

**DOI:** 10.3390/nu10091202

**Published:** 2018-09-01

**Authors:** Jayme L. Workinger, Robert. P. Doyle, Jonathan Bortz

**Affiliations:** 1 Human Nutrition and Pharma, Balchem Corporation, 52 Sunrise Park Road, New Hampton, NY 10958, USA; jbortz@balchem.com; 2 Department of Chemistry, Center for Science and Technology, Syracuse University, 111 College Place, Syracuse, NY 13244, USA; rpdoyle@syr.edu

**Keywords:** magnesium, magnesium deficiency, magnesium absorption, magnesium sampling, magnesium assays, magnesium status

## Abstract

Magnesium is a critical mineral in the human body and is involved in ~80% of known metabolic functions. It is currently estimated that 60% of adults do not achieve the average dietary intake (ADI) and 45% of Americans are magnesium deficient, a condition associated with disease states like hypertension, diabetes, and neurological disorders, to name a few. Magnesium deficiency can be attributed to common dietary practices, medications, and farming techniques, along with estimates that the mineral content of vegetables has declined by as much as 80–90% in the last 100 years. However, despite this mineral’s importance, it is poorly understood from several standpoints, not the least of which is its unique mechanism of absorption and sensitive compartmental handling in the body, making the determination of magnesium status difficult. The reliance on several popular sample assays has contributed to a great deal of confusion in the literature. This review will discuss causes of magnesium deficiency, absorption, handling, and compartmentalization in the body, highlighting the challenges this creates in determining magnesium status in both clinical and research settings.

## 1. Introduction

Magnesium is a critical mineral in the human body governing the activity of hundreds of enzymes encompassing ~80% of known metabolic functions [1,2,3,4]. Despite the importance of magnesium, it remains one of the least understood and appreciated elements in human health and nutrition. It is currently estimated that 45% of Americans are magnesium deficient and 60% of adults do not reach the average dietary intake (ADI) [5,6,7,8]. A daily intake (DI) of 3.6 mg/kg is necessary to maintain magnesium balance in humans under typical physiological conditions, with the ADI for adults estimated at between 320 to 420 mg/day (13–17 mmol/day) [9,10]. 

The high rate of magnesium deficiency now postulated [5,6,7,8] can be attributed in part to a steady decline in general magnesium content in cultivated fruits and vegetables, a reflection of the observed depletion of magnesium in soil over the past 100 years [11,12,13]. A report to Congress was already sounding the alarm as far back as the 1930s, pointing out the paucity of magnesium, and other minerals, in certain produce [14].

This loss of mineral content across “healthy” food choices has been compounded by a historical rise in the consumption of processed food, which has been shown to impede magnesium absorption and contribute to the current state of magnesium deficiency (defined by serum blood levels, “normal” being considered as 0.7–1 mmol/L and hypomagnesaemia as <0.7 mmol/L) [15,16,17,18,19]. Given the role of magnesium in calcium and potassium transport, cell signaling, energy metabolism, genome stability, DNA repair and replication, it is not surprising that hypomagnesaemia is now associated with many diseases including hypertension, coronary heart disease, diabetes, osteoporosis, and several neurological disorders [1,2,4,20,21,22,23]. 

**Figure 1:**
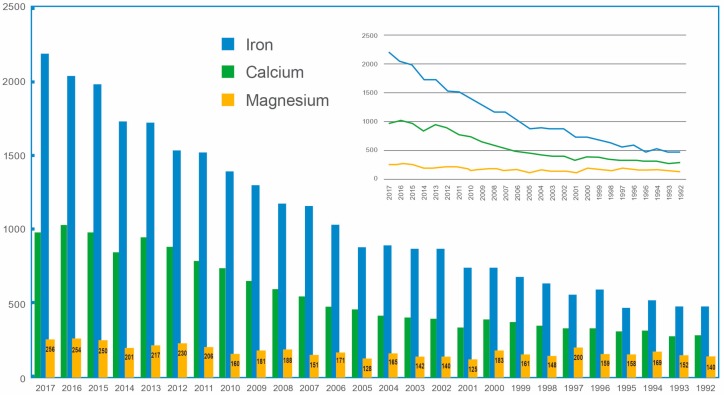
Number of basic and clinical research papers published (Y-axis) as screened using Web of Science [v.5.28.1] under the search terms “magnesium deficiency” (yellow), “calcium deficiency” (green) or “iron deficiency” (blue) (performed 4 May 2018) over the past 25 years (X-axis; 2017–1992). (Inset) Trend lines show the relatively flat research output on magnesium deficiency relative to calcium and iron.

Despite its importance to human health, magnesium remains one of least investigated macro minerals, and while it is getting more attention, this still pales in comparison to the level of investigation into other macronutrients such as calcium or iron (Figure 1). The root cause of this oversight likely lies in the fact that iron and calcium deficiency can be diagnosed through a variety of clinically well recognized associated signs and symptoms, and readily supported by commonly used, and clinically validated, diagnostic assays available for verification [24,25,26]. This tie-in is not the case however, for magnesium, where deficiency does not present with unique and identifiable clinical manifestations. Furthermore, even if clinical signs and symptoms are present, they are overshadowed by or taken to be the result of common co-morbidities such as diabetes and cardiovascular disease. The lack of a standardized laboratory test that accurately describes the status of magnesium [27] remains one of the most vexing challenges associated with the magnesium field, and contributes to the relative anonymity of magnesium compared to other macronutrients, which in turn, further contributes to magnesium deficiency and its sequelae.

Moving forward, it is clear that there will be an important role to play for magnesium supplementation across, and within, certain populations. The key to unlocking the benefits of magnesium will be to understand the factors contributing to inadequate dietary intake, including the complexity of absorption, secretion, and reabsorption, and to address the challenges of representative compartment analytics. These factors make most human clinical magnesium supplementation studies are difficult to extrapolate and interpret accurately, leading to magnesium research being described as, “Far from complete and the conclusions that have been drawn are far from clear.” [28].

### Causes of Magnesium Deficiency

Despite the importance of magnesium to human health and wellness, 60% of people do not meet the recommended DI of 320 mg/day for woman and 420 mg/day for men, with 19% not obtaining even half of the recommended amount [5,6,29]. Magnesium dietary deficiency can be attributed not just to poor mineral intake due to modern diets, but historical farming practices may play a significant role as well. The highest food sources of magnesium are leafy greens (78 mg/serving), nuts (80 mg/serving), and whole grains (46 mg/serving), none of which individually have a high percentage of the recommended dietary allowance (RDA) of magnesium or are eaten consistently or sufficiently for adequate magnesium intake [10,15,30]. Increasing demand for food has caused modern farming techniques to impact the soil’s ability to restore natural minerals such as magnesium. In addition, the use of phosphate-based fertilizers has resulted in the production of aqueously insoluble magnesium phosphate complexes, for example, further depriving the soil of both components [31].

**Figure 2:**
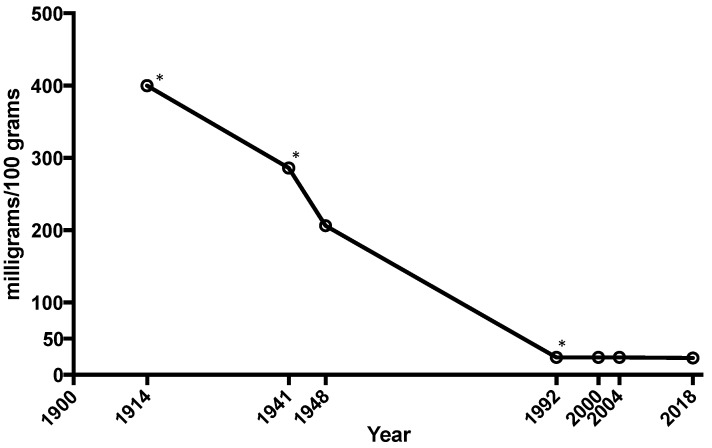
The average mineral content of calcium, magnesium, and iron in cabbage, lettuce, tomatoes, and spinach has dropped 80–90% between 1914 and 2018 [30,34,35,36,37]. Asterisks indicate numbers could not be independently verified.

Many fruits and vegetables have lost large amounts of minerals and nutrients in the past 100 years with estimates that vegetables have dropped magnesium levels by 80–90% in the U.S. (Figure 2) and the UK [11,12,13,32,33]. It is important to note that the USDA mineral content of vegetables and fruits has not been updated since 2000, and perhaps even longer, given that the data for 1992 was not able to be definitively confirmed for this review. The veracity of the mineral content to support the claim of demineralization of our food sources should be verified, particularly since farming methods and nutrient fertilization has undoubtedly advanced in the last 50 years. Hence, there is a clear need for a new initiative to study the current mineral content in vegetables and fruits grown in selective markets to get a current and validated assessment of the mineral and nutrient value of commonly consumed fruit and vegetable staples.

Modern dietary practices are now estimated to consist of up to 60% processed foods [38]. Processing techniques, such as grain bleaching and vegetable cooking, can cause a loss of up to 80% of magnesium content [39]. Beverages, such as soft drinks, which contain high phosphoric acid, along with a low protein diet (<30 mg/day), and foods containing phytates, polyphenols and oxalic acid, such as rice and nuts, all contribute to magnesium deficiency due to their ability to bind magnesium to produce insoluble precipitates, thus negatively impacting magnesium availability and absorption [40,41,42,43]. Magnesium in drinking water contributes to about 10% of the ADI [44], however, increased use of softened/purified tap water can contribute to magnesium deficiency due to the filtering or complexation of the metal [45]. In addition, fluoride, found in 74% of the American population’s drinking water, with ~50% of drinking water having a concentration of 0.7 mg/L, prevents magnesium absorption through binding and production of insoluble complexes [46,47,48]. Ingestion of caffeine and alcohol increase renal excretion of magnesium causing an increase in the body’s demand [49,50]. Common medications can also have a deleterious effect on magnesium absorption such as antacids (e.g., omeprazole), due to the increase in gastrointestinal (GI) tract pH (see Section 2.5) [51,52], antibiotics (e.g., ciprofloxacin) [53], and oral contraceptives due to complexation [54,55], and diuretics (e.g., furosemide and bumetanide), due to an increase in renal excretion (see Section 2.6) [56,57]. 

## 2. Magnesium Absorption

### 2.1. Anatomic Considerations

**Figure 3:**
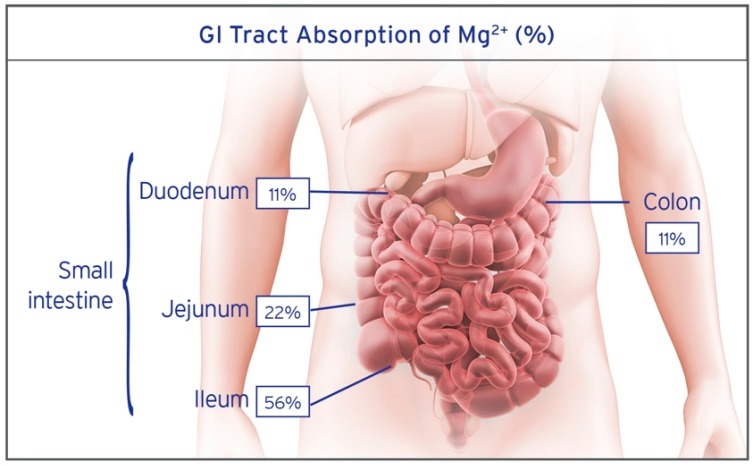
Percentage of magnesium absorption in the GI tract. The majority of magnesium is absorbed in the distal portion of the small intestine. The ileum absorbs 56%, the jejunum 22%, the duodenum 11%, and colon 11% [3,58].

Unlike other minerals, magnesium can be absorbed along the entire length of the gastrointestinal tract. However, different segments contribute unequally to the overall absorption of dietary magnesium. Due to the complex nature of magnesium absorption, segments of the GI tract can vary in their contribution to absorption, however, under normal physiologic conditions the general guide is the duodenum absorbs 11%, the jejunum 22%, the ileum 56%, and the colon 11% (Figure 3) [3,58].

### 2.2. Absorption

**Figure 4:**
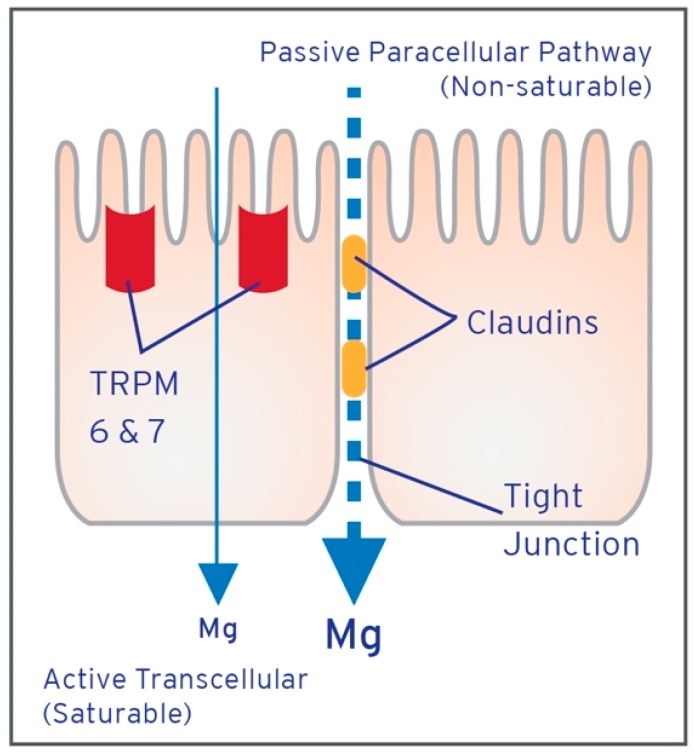
Magnesium absorption in the intestine. Magnesium is absorbed through either a saturable transcellular pathway (**left**) in which TRPM6 and TRPM7 actively transport magnesium into the GI epithelial cells, which is effluxed through a Na^+^/Mg^2+^ exchanger and/or a paracellular pathway (**right**) where magnesium transverses the tight junctions of the intestinal epithelium, assisted by magnesium associated claudin proteins.

Two transport systems, one passive and one active, are known to be responsible for magnesium uptake (Figure 4). At lower intestinal magnesium concentrations, a transcellular and saturable transport mechanism predominates and relies on an active transporter [20,59,60], Transient Receptor Potential Channel Melastatin members (TRPM6 and TRPM7), which possess unusual properties designed to strip away the hydration shell of magnesium (see Section 2.3) [61,62,63,64,65,66,67]. This active transport occurs predominantly in the distal small intestine and colon, and due to saturability is only responsible for 10–20% of total magnesium absorbed [68]. Additionally, active transport can increase magnesium absorption, typically at 30–50% [69] of ingested magnesium, up to 80%, for periods of time during lower luminal concentrations [70,71]. TRPM6 and TRPM7 have high sensitivity to intracellular magnesium levels causing inhibition and saturation of transcellular transport at higher magnesium concentrations resulting in magnesium absorption being dominated by paracellular transport [64,67].

Passive paracellular diffusion occurs in the small intestine and because it is non-saturable, is responsible for 80–90% of overall magnesium absorption [20,58,60,72]. The driving force behind this passive transport is a high luminal concentration, ranging between 1 and 5 mmol/L, which contributes to an electrochemical gradient and solvent drag of magnesium through the tight junctions between intestinal enterocytes [59,73]. 

**Figure 5:**
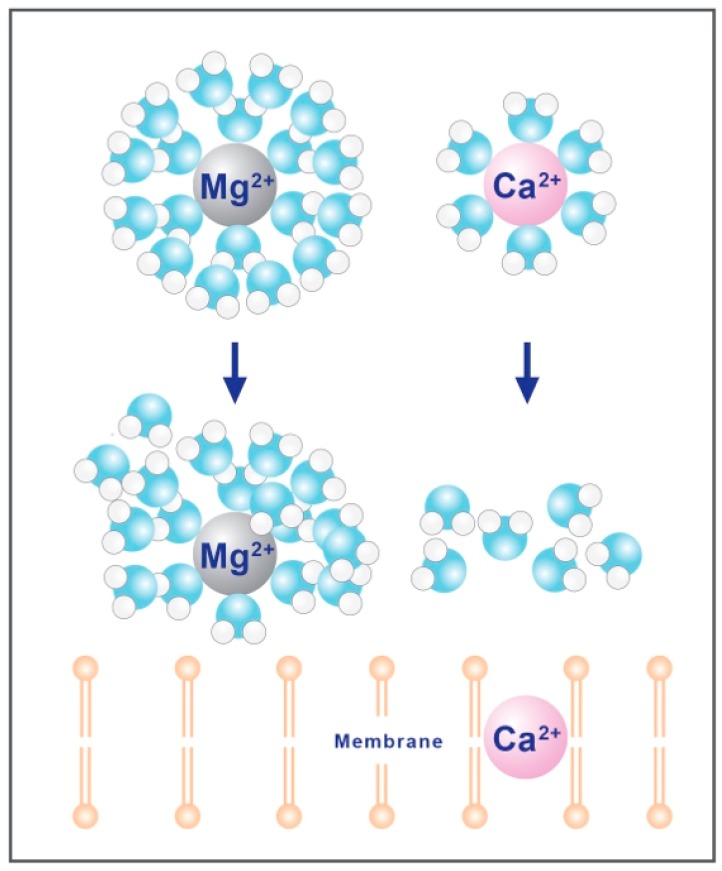
Hydration shells of both magnesium and calcium. The hydrated radius of magnesium is >400 times larger than its dehydrated radius, which is much more prominent than calcium (~25-fold difference) [83,84]. This increase in radius, unlike calcium, prevents magnesium from passing through narrow ion channels.

The distal jejunum and ileum have relatively low expression of certain tightening claudin proteins (1, 3, 4 and 5) [74,75], the integral membrane proteins of tight junctions, which allow a higher permeability, and hence, higher magnesium transport [75,76,77]. Claudins are also known to form paracellular channels, in monomeric or heteromeric combinations, which can electively transport ions such as calcium and magnesium. Studies have shown that when expression of claudins 2, 7 and 12 (all highly expressed in the small intestine) [75,76] was decreased, magnesium paracellular transport was also decreased, indicating that certain claudins play a significant role in passive magnesium transport and absorption [78,79]. Claudins 16 and 19 have been shown to be involved in magnesium reabsorption in the kidney but are not expressed in the GI tract [80]. A series of in vitro experiments, designed to explore the involvement of active (solvent drag, voltage dependent or transcellular transport) and passive paracellular transport mechanisms of magnesium absorption, showed that the paracellular passive pathway was mainly mediated by claudin proteins at the tight junctions, which was attributed to their ability to remove the hydration shell of magnesium (Figure 5) [78,79,81]. 

Claudins are a large family of proteins and the identification, localization, and function of these integral membrane proteins is part of an emerging science, which at present only allows a glimpse into their role in mineral transport in general, and magnesium transport in particular [75,76,77,82].

### 2.3. Hydration Shell

Magnesium is a divalent cation, which plays a critical role in how the mineral is absorbed [83,84]. Magnesium is the most densely charged of all the biological cations, due to a high charge to radius ratio, resulting in high hydration energy for the Mg^2+^ cation [83]. This hydration energy results in tight coordination with a double layer of water molecules, increasing the hydrodynamic radius 400 times that of the dehydrated radius [83,85], resulting in an aquated cation that is too large to transverse typical ion channels (Figure 5) [86]. The removal of the hydration shell around magnesium is a precondition for absorption and can be accomplished by both TMPR6 and TMPR7 and the magnesium associated paracellular claudins [64,67,87,88].

### 2.4. Distribution in the Human Body

**Figure 6:**
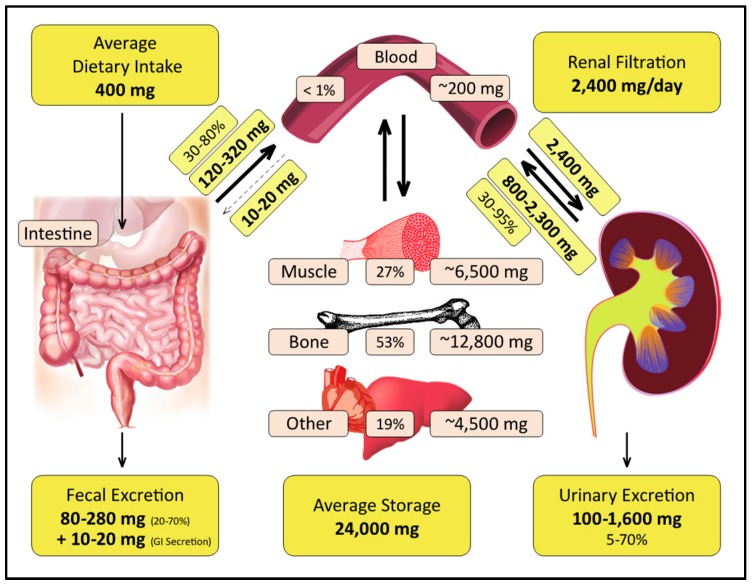
Magnesium homeostasis. Dietary magnesium can be absorbed along the entire length of the GI tract and into the blood but can also be excreted in feces (between 20% and 70% of the ingested amount) [69]. Once in the blood, magnesium is quickly taken up into tissues with muscle containing 27%, bone 53%, and other tissues holding 19% [3,58,93]. Blood and tissue magnesium are in a constant state of exchange and the kidney, which can filter up to 2400 mg of magnesium per day [94] (or 10% of average magnesium content in an adult [95]) can excrete between 5% and 70% of that magnesium depending on multiple variables.

Once magnesium is absorbed it is distributed throughout the body for use and storage. Only 0.8% of magnesium is found in blood with 0.3% in serum and 0.5% in erythrocytes, with a typical total magnesium serum concentration between 0.65–1.0 mmol/L [89,90]. The rest is distributed in soft tissue (19%), muscle (27%), and bone (53%) (Figure 6) [89,90,91]. Up to one-third of the magnesium stored in bone is exchangeable [92], and while the total amount of magnesium stored in bone can change with age, bone remains the most significant area of stored and exchangeable magnesium.

### 2.5. Factors That Influence Magnesium Absorption

Magnesium concentration within the GI tract is a key driver of how and which of the two transport systems become engaged in magnesium absorption. Active transport in the colon dominates absorption at lower magnesium concentrations but becomes saturated when luminal amounts are between 125 and 250 mg [70,72]. When luminal amounts reach ≥250 mg the absorption mechanism changes and is governed by passive transport in the distal small bowel [70,72].

That being said, the solubility of the magnesium form (inorganic salt, organic salt, chelate, etc.) is an important factor, with increased solubility correlating with increased absorption. The pH of the GI tract can impact how soluble the magnesium form is, with a lower pH increasing magnesium solubility [96,97]. This can make magnesium absorption increasingly difficult as it travels down the small intestine with pH steadily increasing to 7.4 in the ileum. In 2005, Coudray et al. showed that magnesium absorption is significantly affected by GI tract pH in rats [97]. The study showed that as pH gradually increased, the solubility of ten magnesium salts (organic and inorganic) gradually decreased from 85% in the proximal intestine to 50% in the distal intestine. Other studies showed that a commonly used proton pump inhibitor, omeprazole, affected passive transport in vitro [78,79]. They showed that omeprazole suppressed passive magnesium absorption by causing luminal acidity to rise above the range (pH 5.5–6.5) in which claudin 7 and 12 expression is optimized, magnesium hydration shell stripping is most effective, and electrostatic coupling between magnesium and the transporter takes place [79]. 

Magnesium absorption is enhanced by factors that contribute to water flow across the intestinal mucosal membrane, such as simple sugars and urea [59,98]. Therefore, meals containing carbohydrates and medium chain fatty acids will increase magnesium uptake but will also increase the demand since magnesium is critical to glucose breakdown and insulin release [99]. Solid meals, by prolonging GI transit time, can also enhance magnesium absorption [100]. Increased dietary fiber intake in the diet (e.g., cellulose, pectin, and inulin) does not appear to affect magnesium status but can increase magnesium excretion in feces [101,102,103]. 

### 2.6. Factors That Affect Magnesium Status 

Renal function is a key player in magnesium homeostasis and filters approximately 2400 mg/day [94], and anywhere between 5% and 70% filtered magnesium may actually be excreted in the urine [89,103,104]. This wide range depends on ever changing variables such as dietary intake, existing magnesium status, mobilization from bone and muscle, and the influence of a variety of hormones (e.g., parathyroid hormone, calcitonin, glucagon) [105,106,107] and medications (e.g., diuretics and certain chemotherapies that can cause abnormally high magnesium excretion) [56,90,104,108]. Renal magnesium wasting can occur in patients who are on long-term diuretic management as well as those with diabetes. The resultant magnesium deficiency leads to higher nutritional requirements and the inevitable increase in magnesium absorption to re-establish homeostasis [109]. 

Gender also contributes to magnesium status as estrogen enhances magnesium utilization, favoring its uptake by soft and hard tissues [110]. Young women have better magnesium retention than young men, and as a result of this, their circulating magnesium levels are lower [111,112], particularly at the time of ovulation or during oral contraceptive use [54,112,113,114,115], when estrogen levels are highest. Consequently, samples taken in a mixed gender population or at time points that do not take this into account could further confound human magnesium studies.

Body mass index (BMI) also may affect magnesium status, particularly in women and children. Patients considered obese (BMI ≥ 30) have been shown to have lower magnesium consumption and reduced magnesium status compared to non-obese age matched controls [116,117,118,119].

## 3. Analytical Challenges in Establishing Magnesium Status

Understanding the relationship between the concentration of an analyte in the compartment being measured (e.g., blood, urine, and epithelial samples) and the status of that analyte in the body, or its relevance in the measured compartment is a fundamental principal that will render an analytical test useful or not. Due to the way in which magnesium is compartmentalized, the typical compartment (blood and urine) analytics may not provide an accurate proxy of magnesium status and will mislead the practitioner.

A literature search identified 54 randomized controlled magnesium supplementation studies (see Methods), and showed that the majority of studies examined blood and urine with only a few examining fecal material, other tissues such as muscle, or from different cell specimens (Table 1).

### 3.1. Blood Levels

The current “normal” range interval of serum magnesium is 0.7–1 mmol/L and was established based on serum magnesium levels gathered by a U.S. study between 1971 and 1974 of presumably healthy individuals aged 1–74 years [120]. Serum changes can be influenced by dietary magnesium intake and albumin levels, but can also be affected by short term changes like day to day and hour to hour variability of the amount of magnesium absorbed and excreted through the kidneys [121]. Blood levels have been shown to increase in response to magnesium supplementation, but this does not signal that complete equilibrium has been established between blood and the nearly 100-fold larger body reservoir of magnesium. In fact, the much larger exchangeable pool of magnesium is more often called upon to augment blood levels to maintain a narrow range preferentially, which is a key reason why blood measurements can easily mask deficiency [122,123].

The tight control of magnesium serum levels, representing only 0.8% of total body stores (see Section 2.4), therefore serves as a poor proxy for the 99.2% of magnesium in other tissues that constitutes the body’s true magnesium status. Furthermore, this narrow serum range feeds the common perception of clinicians that magnesium levels rarely fluctuate, and therefore, are not indicative of the condition for which the blood tests are ordered. Therefore, practitioners are apt to order blood tests for magnesium infrequently, if at all, and if a magnesium level is in the patient chart, it is more often as part of a blood test panel and not purposely ordered to determine the magnesium status [89,124,125,126]. This contributes significantly to magnesium deficiency not being recognized as a modifiable nutritional intervention, and magnesium in general, being the neglected mineral that it is. 

Red blood cells’ (RBC; erythrocyte and monocyte) magnesium levels are often cited as preferable to serum or plasma levels due to their higher magnesium content (0.5% vs. 0.3%, respectively). Some RBC studies report correlation to magnesium status particularly when subjects are placed on long-term (~3 months) magnesium replete or deplete diets. However, most studies using RBC magnesium endpoints do not satisfy this long-term design and have not been performed in nearly enough randomized clinical studies to be considered sufficiently robust or reliable (Table 1) [127,128,129]. In addition, the majority of RBC studies do not validate the method through inter-compartmental sampling (e.g., urine and muscle), challenging the claim that this test is a reliable representation of the large magnesium pool.

### 3.2. Urine Levels

Due to the large amount of magnesium filtered and the variable degree of reabsorption and secretion (see Section 2.6), magnesium levels in the urine do not correlate with either the amount of magnesium ingested or the magnesium status in the body. Therefore, despite their frequent use in many published clinical studies (Table 1) [6,130], they should be regarded critically in most clinical and research settings due to the wide fluctuation of renal magnesium reabsorption and excretion.

An epidemiologic study linking magnesium status with risk of heart disease highlighted the poor correlation between urine and blood results and called out the inconsistent results from many previous studies [186,187], even though 24 h urine analyses may still serve some useful function in population based epidemiological studies. The biological variation of magnesium status in smaller cohorts, however, has been highlighted in a study with 60 healthy males in which a within-subject variation of 36% and a between-subject variation of 26% was demonstrated when measuring the 24 h urinary magnesium excretion [187]. The same can be said about fecal magnesium levels, which require 3–7 days collection and are notoriously unpopular with researchers and subjects [69,188]. 

A more complicated method of determining magnesium status relies on intravenous magnesium loading followed by a 24 h urine collection, ostensibly to measure what percentage of administered dose is retained, from which an assessment of magnesium status can be derived. This retention test relies heavily on the reliability and standardization of the 24 h urine measurement, which is not uniformly accepted [125,189,190,191,192,193]. Additionally, this test is costly, more suitable for research units and impractical for most clinical settings.

### 3.3. Oral Sampling

Energy-dispersive X-ray analysis of magnesium in sublingual cells reports correlation between intracellular magnesium levels in sublingual cells and atrial cell biopsies from subjects undergoing open heart surgery in a small single cohort [194]. However, to our knowledge, this method has not been validated for application as a reliable and indicative of magnesium status in a broader context, beyond a single disease state cohort study. So too, saliva levels have not been adequately correlated with other conventional measurements, and therefore, to date, lack the requisite robustness to be considered as an improvement to assays of blood or urine [155]. 

### 3.4. Magnesium Isotopes 

In recognition of the meaningful exchange of endogenous magnesium between physiologic compartments, and the high degree of biological variability in typical analytic measurements, some researchers maintain that the only reliable way of measuring the disposition of exogenous magnesium is by using isotopic labels [97,195,196,197,198,199,200]. A radioisotope, ^28^magnesium, has been used previously in magnesium research but it does not make an ideal nucleotide because its half-life (*t*_1/2_ = 21 h) [201,202,203] does not match the long biological half-life of magnesium (~1000 h) [201]. Therefore, ^28^magnesium is not commonly used in current research [128]. 

Stable isotopes retain all chemical characteristics of an element while being distinguishable from the endogenous elements within the body. This allows for a means of tracking the fate of an exogenously administered “dose” of the element upon ingestion or injection into the body without the harmful emissions associated with radioisotopes. Stable isotopes can be useful tools, particularly in nutritional research, because of the ability to use them in most populations (including small children and pregnant women) and more than one isotope can be used in a study to follow uptake and distribution of different forms of a nutrient. 

However, stable magnesium isotopes have proven to be difficult to use because truly low-abundance stable magnesium isotopes do not exist, and therefore, provide significant background noise in the assays. There are three stable magnesium isotopes; ^25^magnesium, which has an abundance of 10%, ^24^magnesium has 79% abundance, and ^26^magesnium has 11% abundance. This means that these isotopes cannot be used in the customary small amounts to provide an adequate isotope signal to indicate magnesium status [84]. Very large amounts of isotope, using more than one isotope or significant enrichment, are needed for these studies, dramatically limiting the available supply and adding significantly to the cost, ultimately leading researchers to use less sophisticated and unreliable methods. 

## 4. Conclusions

An argument can be made for revisiting the accepted ranges of diagnostic tests to capture clinical or other biologic consequences that lie within the currently accepted ranges of normal. Even though this has been suggested in a recent review [6], this approach is likely to be more impactful in large population studies (that have not been undertaken in the U.S. for more than 40 years) than provide pinpoint guidance to diagnose and manage magnesium deficiency in the individual. The multiple factors affecting magnesium status (e.g., dietary intake, luminal concentration, GI pH, weight and gender) in conjunction with the high degree of inter- and intra-variability of intestinal, renal, and tissue handling make an individual diagnosis extremely challenging for the clinician. 

Until a commercially viable and unambiguous magnesium deficiency biomarker is identified and validated, it is worth exploring an alternative approach to diagnosing magnesium deficiency. A patient with dietary risk factors (e.g., high soda, coffee, and processed food ingestion); using medications known to affect magnesium (e.g., diuretics, antacids, oral contraceptives); with disease states (e.g., ischemic heart disease, diabetes, and osteoporosis); with clinical symptoms (e.g., leg cramps, sleep disorder, and chronic fatigue); or with Metabolic Syndrome (Table 2) should prompt the practitioner to measure serum and/or 24 h urine for magnesium, bearing in mind that it is quite likely that results from these laboratory tests may read within the reference range (0.75–0.85 mmol/L in the case of serum magnesium) [204]. 

It has further been suggested that if serum magnesium is below 0.85 mmol/L and urinary excretion is below 80 mg/day, it is appropriate to consider magnesium related co-morbidities and risk factors for magnesium deficiency when considering whether a state of magnesium deficiency exists [204]. This could warrant a medication change or dietary recommendations to increase intake of raw vegetables with higher magnesium content and reducing soda and processed food consumption with low or no magnesium and/or recommending magnesium supplements.

This new approach may further sensitize the clinician to the limitations of diagnostic tests and the need to incorporate risk and clinical considerations into the treatment paradigm. By way of suggestion, certain conditions may be regarded as “major” diagnostic criteria (e.g., diuretic use, ischemic heart disease, and high processed food and/or soda intake) and others as “minor” criteria (e.g., sleep disorder or BMI) (Table 2).

A clinician may recognize a patient at risk for magnesium deficiency with one major criterion and two or more minor criteria, or two major criteria and no minor criteria, and so forth. The parameters of such a system is beyond the scope and authority of this review, with Table 2 being illustrative, but this could be entertained as a new way to look at a serious essential nutrient deficiency that is all but ignored because of the pitfalls of the analytic methods that are peculiar to this most important mineral. 

## Figures and Tables

**Table 1:** Magnesium clinical trial studies by year with method of determining magnesium status indicated. Expanded from Zhang et al. [130].

Study	Blood		Urine		Intracellular				Fecal	Tissue		Challenge Studies	
	Serum	Plasma	24 h	NS	RBC	WBC	SL	Other		Muscle	Other	Balance	Retention
1. Zemel, 1990, USA [131]			χ		χ								
2. Facchinetti, 1991, Italy [132]		χ			χ	χ							
3. Desbiens, 1992, USA [133]	χ												
4. Ferrara, 1992, Italy [134]	χ		χ										
5. Bashir, 1993, USA [135]	χ		χ										
6. Plum-Wirell, 1994, Sweden [136]	χ		χ							χ			
7. Witteman, 1994, Netherlands [137]	χ			χ									
8. Eibl, 1995, Austria [138]	χ			χ									
9. Eriksson, 1995, Finland [139]		χ											
10. Itoh, 1996, Japan [140]	χ		χ										
11. Sanjuliani, 1996, Brazil [141]					χ								
12. Costello, 1997, USA [142]	χ		χ		χ								
13. Sacks, 1997, USA [143]				χ									
14. de Valk, 1998, Netherlands [144]		χ	χ		χ								
15. Lima, 1998, Brazil [145]		χ		χ		χ							
16. Walker, 1998, UK [146]			χ										
17. Weller, 1998, Germany [147]	χ		χ		χ	χ				χ			
18. Hagg, 1999, Sweden [148]	χ			χ									
19. Wary, 1999, French [149]		χ	χ		χ					χ	χ		
20. Zorbas, 1999, Greece [150]		χ		χ					χ			χ	
21. Schechter, 2000, USA [151]			χ				χ						
22. Walker, 2002, UK [152]				χ									
23. Mooren, 2003, Germany [153]					χ								
24. Rodriguez-Moran, 2003, Mexico [154]		χ											
25. Walker, 2003, UK [155]		χ	χ					χ					
26. Závaczki, 2003, Hungary [156]	χ												
27. De Leeuw, 2004, Belgium [157]	χ				χ								
28. Pokan, 2006, USA [158]							χ						
29. Rodríguez, 2008, Mexico [159]	χ												
30. Almoznino-Sarafian, 2009, Israel [160]	χ							χ					
31. Lee, 2009, South Korea [161]	χ										χ		
32. Romero, 2009, Mexico [162]	χ												
33. Aydın, 2010, Turkey [163]	χ												
34. Kazaks, 2010, USA [164]	χ				χ								χ
35. Nielsen, 2010, USA [165]	χ		χ		χ								
36. Zorbas, 2010, Greece [166]		χ		χ					χ	χ			
37. Chacko, 2011, USA [167]	χ												
38. Romero, 2011, Mexico [168]	χ												
39. Esfanjani, 2012, Iran [169]	χ												
40. Laecke, 2014, Belgium [170]	χ			χ									
41. Cosaro, 2014, Italy [171]		χ		χ				χ					
42. Rodriguez, 2014, Mexico [172]	χ												
43. Setaro, 2014, Brazil [173]	χ												
44. Navarrete-Cortes, 2014, Mexico [174]	χ		χ										
45. Guerrero-Romero, 2015, Mexico [175]	χ												
46. Park, 2015, USA [176]	χ												
47. Baker, 2015, USA [177]	χ												
48. Joris, 2016, Netherlands [178]	χ		χ										
49. Terink, 2016, Netherlands [179]		χ											
50. Moradian, 2017, Iran [180]	χ												
51. Rajizadeh, 2017, Iran [181]	χ												
52. Cunha, 2017, Brazil [182]					χ								
53. Bressendorff, 2017, Denmark/Norway [183]	χ		χ										
54. Bressendorff, 2017, Denmark [184]	χ		χ				χ						
55. Toprak, 2017, Turkey [185]	χ												
**Total**	**35**	**11**	**16**	**10**	**12**	**3**	**3**	**3**	**2**	**4**	**2**	**1**	**1**

RBC: red blood cells; WBC: white blood cells; SL: sublingual cells; NS: not specified or not 24 h collection.

**Table 2:** Suggested illustrative criteria for assessment of magnesium deficiency.

Category	Risk Factor	Criterion
Disease	Diabetes [4], Heart disease [22]	Major
	Osteoporosis [26]	Minor
Diet	Soda [41], Processed Foods [39]	Major
	Coffee [50], Alcohol [49], Protein [42]	Minor
Medication	Diuretics [57], Antacids [51]	Major
	Oral contraceptives [55], Antibiotics [53]	Minor
Clinical History	Leg Cramps [205]	Major
	Sleep Disorder [206], Fibromyalgia [207], Chronic fatigue [208]	Minor
Metabolic Status	Metabolic Syndrome [209]	Major
	BMI > 30 [117]	Minor

## Methods

Searches were conducted in MEDLINE up to 30 May 2018. The words “magnesium”, “supplementation”, “intervention,” “depletion”, “randomized controlled trial”, “randomized clinical trial”, “randomized trial”, “controlled trial”, and “clinical trial” were used in the searches.

## References

[1] Geiger H., Wanner C. (2012). Magnesium in disease. Clin. Kidney J..

[2] Volpe S.L. (2013). Magnesium in Disease Prevention and Overall Health. Adv. Nutr..

[3] McCarthy J.T., Kumar R. (1999). Atlas of Diseases of the Kidney: Divalent Cation: Magnesium.

[4] De Baaij J.H.F., Hoenderop J.G.J., Bindels R.J.M. (2015). Magnesium in Man: Implications for Health and Disease. Physiol. Rev..

[5] Fulgoni V.L., Keast D.R., Bailey R.L., Dwyer J. (2011). Foods, Fortificants, and Supplements: Where Do Americans Get Their Nutrients?. J. Nutr..

[6] Costello R.B., Elin R.J., Rosanoff A., Wallace T.C., Guerrero-Romero F., Hruby A., Lutsey P.L., Nielsen F.H., Rodriguez-Moran M., Song Y. (2016). Perspective: The Case for an Evidence-Based Reference Interval for Serum Magnesium: The Time Has Come12345. Adv. Nutr..

[7] Rosenstein D.L., Ryschon T.W., Niemela J.E., Elin R.J., Balaban R.S., Rubinow D.R. (1995). Skeletal muscle intracellular ionized magnesium measured by 31P-NMR spectroscopy across the menstrual cycle. J. Am. Coll. Nutr..

[8] What We Eat in America, NHANES 2011–2012, Day 1 Food and Supplement Intake Data. https://www.ars.usda.gov/ARSUserFiles/80400530/pdf/1112/Table_37_SUP_GEN_11.pdf.

[9] Institute of Medicine (US) Standing Committee on the Scientific Evaluation of Dietary Reference Intakes (1997). Dietary Reference Intakes for Calcium, Phosphorus, Magnesium, Vitamin D, and Fluoride.

[10] (2018). Magnesium. Office of Dietary Supplements: National Institutes of Health. http://ods.od.nih.gov/factsheets/folate.

[11] Fardet A. (2013). Food and Nutrition Sciences—Open Special Issues: Public Health Nutrition Initiatives. Food Nutr. Sci..

[12] Davis D.R. (2009). Declining Fruit and Vegetable Nutrient Composition: What Is the Evidence?. HortScience.

[13] Guo W., Nazim H., Liang Z., Yang D. (2016). Magnesium deficiency in plants: An urgent problem. Crop J..

[14] Senate Document 264, 74th Congress, 2nd Session, 5 June 1936. https://www.prismnet.com/~lenb/centurynutrition/senate264.htm.

[15] Current Eating Patterns in the United States—2015–2020 Dietary Guidelines. https://health.gov/dietaryguidelines/2015/guidelines/chapter-2/current-eating-patterns-in-the-united-states/.

[16] Cordain L., Eaton S.B., Sebastian A., Mann N., Lindeberg S., Watkins B.A., O’Keefe J.H., Brand-Miller J. (2005). Origins and evolution of the Western diet: Health implications for the 21st century. Am. J. Clin. Nutr..

[17] Bowman S.A., Gortmaker S.L., Ebbeling C.B., Pereira M.A., Ludwig D.S. (2004). Effects of Fast-Food Consumption on Energy Intake and Diet Quality Among Children in a National Household Survey. Pediatrics.

[18] Paeratakul S., Ferdinand D.P., Champagne C.M., Ryan D.H., Bray G.A. (2003). Fast-food consumption among US adults and children: Dietary and nutrient intake profile. J. Am. Diet. Assoc..

[19] Steele E.M., Popkin B.M., Swinburn B., Monteiro C.A. (2017). The share of ultra-processed foods and the overall nutritional quality of diets in the US: Evidence from a nationally representative cross-sectional study. Popul. Health Metr..

[20] de Baaij J.H.F., Hoenderop J.G.J., Bindels R.J.M. (2012). Regulation of magnesium balance: Lessons learned from human genetic disease. Clin. Kidney J..

[21] Al-Ghamdi S.M.G., Cameron E.C., Sutton R.A.L. (1994). Magnesium Deficiency: Pathophysiologic and Clinical Overview. Am. J. Kidney Dis..

[22] DiNicolantonio J.J., O’Keefe J.H., Wilson W. (2018). Subclinical magnesium deficiency: A principal driver of cardiovascular disease and a public health crisis. Open Heart.

[23] Stritt S., Nurden P., Favier R., Favier M., Ferioli S., Gotru S.K., van Eeuwijk J.M.M., Schulze H., Nurden A.T., Lambert M.P. (2016). Defects in TRPM7 channel function deregulate thrombopoiesis through altered cellular Mg^2+^ homeostasis and cytoskeletal architecture. Nat. Commun..

[24] Lopez A., Cacoub P., Macdougall I.C., Peyrin-Biroulet L. (2016). Iron deficiency anaemia. Lancet.

[25] Weaver C.M., Peacock M. (2011). Calcium. Adv. Nutr..

[26] Nordin B.E.C. (1960). Osteomalacia, Osteoporosis and Calcium Deficiency. Clin. Orthop. Relat. Res..

[27] Reinhart R.A. (1988). Magnesium Metabolism: A Review with Special Reference to the Relationship Between Intracellular Content and Serum Levels. Arch. Intern. Med..

[28] Di Silvestro R. (2013). Current Research on Comparative Utility of Magnesium Supplements. Nat. Prod. Insider.

[29] King D.E., Mainous A.G., Geesey M.E., Woolson R.F. (2005). Dietary magnesium and C-reactive protein levels. J. Am. Coll. Nutr..

[30] USDA, Agricultural Research Service USDA National Nutrient Database for Standard Reference, Release 28. https://www.ars.usda.gov/northeast-area/beltsville-md-bhnrc/beltsville-human-nutrition-research-center/nutrient-data-laboratory/docs/usda-national-nutrient-database-for-standard-reference/.

[31] Schulze-Rettmer R. (1991). The Simultaneous Chemical Precipitation of Ammonium and Phosphate in the form of Magnesium-Ammonium-Phosphate. Water Sci. Technol..

[32] Davis D.R., Epp M.D., Riordan H.D. (2004). Changes in USDA food composition data for 43 garden crops, 1950 to 1999. J. Am. Coll. Nutr..

[33] Mayer A. (1997). Historical changes in the mineral content of fruits and vegetables. Br. Food J..

[34] Beeson K.C. (1941). The Mineral Composition of Crops with Particular Reference to the Soils in Which They Were Grown: A Review and Compilation.

[35] Firman B. (1948). Ash and Mineral Cation Content of Vegetables. Soil Sci. Soc. Am. Proc..

[36] Lindlahr H. (1914). Nature Cure.

[37] USDA, Agricultural Research Service USDA National Nutrient Database for Standard Reference, Release 13. https://www.ars.usda.gov/northeast-area/beltsville-md-bhnrc/beltsville-human-nutrition-research-center/nutrient-data-laboratory/docs/usda-national-nutrient-database-for-standard-reference/.

[38] Steele E.M., Baraldi L.G., da Costa Louzada M.L., Moubarac J.-C., Mozaffarian D., Monteiro C.A. (2016). Ultra-processed foods and added sugars in the US diet: Evidence from a nationally representative cross-sectional study. BMJ Open.

[39] Devika S.J., Tanumihardjo S.A. (2016). Effects of Different Processing Methods on the Micronutrient and Phytochemical Contents of Maize: From A to Z. Compr. Rev. Food Sci. Food Saf..

[40] Bohn T. (2008). Dietary Factors Influencing Magnesium Absorption in Humans. Curr. Nutr. Food Sci..

[41] Philipp Schuchardt J., Hahn A. (2017). Intestinal Absorption and Factors Influencing Bioavailability of Magnesium—An Update. Curr. Nutr. Food Sci..

[42] Schwartz R., Walker G., Linz M.D., MacKellar I. (1973). Metabolic responses of adolescent boys to two levels of dietary magnesium and protein. I. Magnesium and nitrogen retention. Am. J. Clin. Nutr..

[43] Bohn T., Davidsson L., Walczyk T., Hurrell R.F. (2004). Phytic acid added to white-wheat bread inhibits fractional apparent magnesium absorption in humans. Am. J. Clin. Nutr..

[44] Marx A., Neutra R.R. (1997). Magnesium in drinking water and ischemic heart disease. Epidemiol. Rev..

[45] Rosborg I., Kozisek F., Ferrrante M. (2015). Health Effects of Demineralization Drinking Water. Drinking Water Minerals and Mineral Balance.

[46] Barker L.K., Duchon K.K., Lesaja S., Robison V.A., Presson S.M. (2017). Adjusted Fluoride Concentrations and Control Ranges in 34 States: 2006–2010 and 2015. J. Am. Water Works Assoc..

[47] Machoy-Mokrzynska A. (1995). Fluoride-Magnesium Interaction. J. Int. Soc. Fluoride Res..

[48] Ersoy I.H., Koroglu B.K., Varol S., Ersoy S., Varol E., Aylak F., Tamer M.N. (2011). Serum copper, zinc, and magnesium levels in patients with chronic fluorosis. Biol. Trace Elem. Res..

[49] Rylander R., Mégevand Y., Lasserre B., Amstutz W., Granbom S. (2001). Moderate alcohol consumption and urinary excretion of magnesium and calcium. Scand. J. Clin. Lab. Investig..

[50] Kynast-Gales S.A., Massey L.K. (1994). Effect of caffeine on circadian excretion of urinary calcium and magnesium. J. Am. Coll. Nutr..

[51] William J.H., Danziger J. (2016). Magnesium Deficiency and Proton-Pump Inhibitor Use: A Clinical Review. J. Clin. Pharmacol..

[52] Begley J., Smith T., Barnett K., Strike P., Azim A., Spake C., Richardson T. (2016). Proton pump inhibitor associated hypomagnesaemia—A cause for concern?. Br. J. Clin. Pharmacol..

[53] Polk R.E. (1989). Drug-drug interactions with ciprofloxacin and other fluoroquinolones. Am. J. Med..

[54] Dante G., Vaiarelli A., Facchinetti F. (2016). Vitamin and mineral needs during the oral contraceptive therapy: A systematic review. Int. J. Reprod. Contracept. Obstet. Gynecol..

[55] Akinloye O., Adebayo T.O., Oguntibeju O.O., Oparinde D.P., Ogunyemi E.O. (2011). Effects of contraceptives on serum trace elements, calcium and phosphorus levels. West Indian Med. J..

[56] Dørup I. (1994). Magnesium and potassium deficiency. Its diagnosis, occurrence and treatment in diuretic therapy and its consequences for growth, protein synthesis and growth factors. Acta Physiol. Scand. Suppl..

[57] Lim P., Jacob E. (1972). Magnesium Deficiency in Patients on Long-Term Diuretic Therapy for Heart Failure. Br. Med. J..

[58] Hardwick L.L., Jones M.R., Brautbar N., Lee D.B. (1990). Site and mechanism of intestinal magnesium absorption. Miner. Electrolyte Metab..

[59] Behar J. (1974). Magnesium absorption by the rat ileum and colon. Am. J. Physiol. Leg. Content.

[60] Kiela P.R., Ghishan F.K., Said H.M. (2018). Molecular Mechanisms of Intestinal Transport of Calcium, Phosphate, and Magnesium. Physiology of the Gastrointestinal Tract.

[61] Schlingmann K.P., Gudermann T. (2005). A critical role of TRPM channel-kinase for human magnesium transport. J. Physiol..

[62] Walder R.Y., Landau D., Meyer P., Shalev H., Tsolia M., Borochowitz Z., Boettger M.B., Beck G.E., Englehardt R.K., Carmi R. (2002). Mutation of TRPM6 causes familial hypomagnesemia with secondary hypocalcemia. Nat. Genet..

[63] Chubanov V., Gudermann T., Schlingmann K.P. (2005). Essential role for TRPM6 in epithelial magnesium transport and body magnesium homeostasis. Pflüg. Arch..

[64] Schlingmann K.P., Waldegger S., Konrad M., Chubanov V., Gudermann T. (2007). TRPM6 and TRPM7—Gatekeepers of human magnesium metabolism. Biochim. Biophys. Acta.

[65] Schmitz C., Perraud A.-L., Johnson C.O., Inabe K., Smith M.K., Penner R., Kurosaki T., Fleig A., Scharenberg A.M. (2003). Regulation of vertebrate cellular Mg^2+^ homeostasis by TRPM7. Cell.

[66] Ryazanova L.V., Rondon L.J., Zierler S., Hu Z., Galli J., Yamaguchi T.P., Mazur A., Fleig A., Ryazanov A.G. (2010). TRPM7 is essential for Mg(2+) homeostasis in mammals. Nat. Commun..

[67] Ryazanova L.V., Dorovkov M.V., Ansari A., Ryazanov A.G. (2004). Characterization of the protein kinase activity of TRPM7/ChaK1, a protein kinase fused to the transient receptor potential ion channel. J. Biol. Chem..

[68] Kayne L.H., Lee D.B. (1993). Intestinal magnesium absorption. Miner. Electrolyte Metab..

[69] Schwartz R., Spencer H., Welsh J.J. (1984). Magnesium absorption in human subjects from leafy vegetables, intrinsically labeled with stable 26Mg. Am. J. Clin. Nutr..

[70] Graham L.A., Caesar J.J., Buegen A.S.V. (1960). Gastrointestinal absorption and excretion of Mg28 in man. Metabolism.

[71] Fine K.D., Santa Ana C.A., Porter J.L., Fordtran J.S. (1991). Intestinal absorption of magnesium from food and supplements. J. Clin. Investig..

[72] Brannan P.G., Vergne-Marini P., Pak C.Y., Hull A.R., Fordtran J.S. (1976). Magnesium absorption in the human small intestine. Results in normal subjects, patients with chronic renal disease, and patients with absorptive hypercalciuria. J. Clin. Investig..

[73] Karbach U. (1989). Cellular-mediated and diffusive magnesium transport across the descending colon of the rat. Gastroenterology.

[74] Lu Z., Ding L., Lu Q., Chen Y.-H. (2013). Claudins in intestines. Tissue Barriers.

[75] Amasheh S., Fromm M., Günzel D. (2010). Claudins of intestine and nephron—A correlation of molecular tight junction structure and barrier function. Acta Physiol..

[76] Günzel D., Yu A.S.L. (2013). Claudins and the Modulation of Tight Junction Permeability. Physiol. Rev..

[77] Capaldo C.T., Nusrat A. (2015). Claudin switching: Physiological plasticity of the Tight Junction. Semin. Cell Dev. Biol..

[78] Thongon N., Krishnamra N. (2011). Omeprazole decreases magnesium transport across Caco-2 monolayers. World J. Gastroenterol..

[79] Thongon N., Krishnamra N. (2012). Apical acidity decreases inhibitory effect of omeprazole on Mg^2+^ absorption and claudin-7 and -12 expression in Caco-2 monolayers. Exp. Mol. Med..

[80] Hou J., Renigunta A., Gomes A.S., Hou M., Paul D.L., Waldegger S., Goodenough D.A. (2009). Claudin-16 and claudin-19 interaction is required for their assembly into tight junctions and for renal reabsorption of magnesium. Proc. Natl. Acad. Sci. USA.

[81] Thongon N., Ketkeaw P., Nuekchob C. (2014). The roles of acid-sensing ion channel 1a and ovarian cancer G protein-coupled receptor 1 on passive Mg^2+^ transport across intestinal epithelium-like Caco-2 monolayers. J. Physiol. Sci..

[82] Krause G., Winkler L., Mueller S.L., Haseloff R.F., Piontek J., Blasig I.E. (2008). Structure and function of claudins. Biochim. Biophys. Acta BBA Biomembr..

[83] Wolf F.I., Cittadini A. (2003). Chemistry and biochemistry of magnesium. Mol. Asp. Med..

[84] Maguire M.E., Cowan J.A. (2002). Magnesium chemistry and biochemistry. Biometals.

[85] Tommaso D.D., Leeuw N.H. (2010). de Structure and dynamics of the hydrated magnesium ion and of the solvated magnesium carbonates: Insights from first principles simulations. Phys. Chem. Chem. Phys..

[86] Moomaw A.S., Maguire M.E. (2008). The Unique Nature of Mg^2+^ Channels. Physiology.

[87] Alexandre M.D., Jeansonne B.G., Renegar R.H., Tatum R., Chen Y.H. (2007). The first extracellular domain of claudin-7 affects paracellular Cl- permeability. Biochem. Biophys. Res. Commun..

[88] Fujita H., Sugimoto K., Inatomi S., Maeda T., Osanai M., Uchiyama Y., Yamamoto Y., Wada T., Kojima T., Yokozaki H. (2008). Tight Junction Proteins Claudin-2 and -12 Are Critical for Vitamin D-dependent Ca2+ Absorption between Enterocytes. Mol. Biol. Cell.

[89] Jahnen-Dechent W., Ketteler M. (2012). Magnesium basics. Clin. Kidney J..

[90] Swaminathan R. (2003). Magnesium Metabolism and its Disorders. Clin. Biochem. Rev..

[91] Vormann J. (2003). Magnesium: Nutrition and metabolism. Mol. Asp. Med..

[92] Wallach S. (1988). Availability of body magnesium during magnesium deficiency. Magnesium.

[93] Elin R.J. (1987). Assessment of magnesium status. Clin. Chem..

[94] Blaine J., Chonchol M., Levi M. (2015). Renal Control of Calcium, Phosphate, and Magnesium Homeostasis. Clin. J. Am. Soc. Nephrol..

[95] Wacker W.E., Parisi A.F. (1968). Magnesium metabolism. N. Engl. J. Med..

[96] Heijnen A.M., Brink E.J., Lemmens A.G., Beynen A.C. (1993). Ileal pH and apparent absorption of magnesium in rats fed on diets containing either lactose or lactulose. Br. J. Nutr..

[97] Coudray C., Rambeau M., Feillet-Coudray C., Gueux E., Tressol J.C., Mazur A., Rayssiguier Y. (2005). Study of magnesium bioavailability from ten organic and inorganic Mg salts in Mg-depleted rats using a stable isotope approach. Magnes. Res..

[98] van der Heijden A., van den Berg G.J., Lemmens A.G., Beynen A.C. (1994). Dietary fructose v. glucose in rats raises urinary excretion, true absorption and ileal solubility of magnesium but decreases magnesium retention. Br. J. Nutr..

[99] Coudray C., Demigné C., Rayssiguier Y. (2003). Effects of Dietary Fibers on Magnesium Absorption in Animals and Humans. J. Nutr..

[100] Sabatier M., Arnaud M.J., Kastenmayer P., Rytz A., Barclay D.V. (2002). Meal effect on magnesium bioavailability from mineral water in healthy women. Am. J. Clin. Nutr..

[101] Behall K.M., Scholfield D.J., Lee K., Powell A.S., Moser P.B. (1987). Mineral balance in adult men: Effect of four refined fibers. Am. J. Clin. Nutr..

[102] Drews L.M., Kies C., Fox H.M. (1979). Effect of dietary fiber on copper, zinc, and magnesium utilization by adolescent boys. Am. J. Clin. Nutr..

[103] Coudray C., Bellanger J., Castiglia-Delavaud C., Rémésy C., Vermorel M., Rayssignuier Y. (1997). Effect of soluble or partly soluble dietary fibres supplementation on absorption and balance of calcium, magnesium, iron and zinc in healthy young men. Eur. J. Clin. Nutr..

[104] Cole D.E., Quamme G.A. (2000). Inherited disorders of renal magnesium handling. J. Am. Soc. Nephrol..

[105] Guerrero-Romero F., Rodríguez-Morán M. (2002). Relationship between serum magnesium levels and C-reactive protein concentration, in non-diabetic, non-hypertensive obese subjects. Int. J. Obes..

[106] Vetter T., Lohse M.J. (2002). Magnesium and the parathyroid. Curr. Opin. Nephrol. Hypertens..

[107] de Rouffignac C., Quamme G. (1994). Renal magnesium handling and its hormonal control. Physiol. Rev..

[108] Oun R., Moussa Y.E., Wheate N.J. (2018). The side effects of platinum-based chemotherapy drugs: A review for chemists. Dalton Trans..

[109] Dirks J.H., Wong N.L.M. (1986). Renal Magnesium Wasting Disorders. Phosphate and Mineral Homeostasis.

[110] Seelig M.S. (1993). Interrelationship of magnesium and estrogen in cardiovascular and bone disorders, eclampsia, migraine and premenstrual syndrome. J. Am. Coll. Nutr..

[111] Jankunas R., Driziene Z., Stakisaitis D., Kuliesiene I. (2001). Gender-dependent Magnesium Urinary Excretion in Healthy Adolescents and Adults. Acta Medica Lituanica.

[112] De I.L., Vansant G., Van L.G. (1992). Magnesium and obesity: Influence of gender, glucose tolerance, and body fat distribution on circulating magnesium concentrations. Magnes. Res..

[113] Muneyyirci-Delale O., Nacharaju V.L., Dalloul M., Altura B.M., Altura B.T. (1999). Serum ionized magnesium and calcium in women after menopause: Inverse relation of estrogen with ionized magnesium. Fertil. Steril..

[114] Grossi E., Castiglioni S., Moscheni C., Antonazzo P., Cetin I., Savasi V.M. (2017). Serum magnesium and calcium levels in infertile women during a cycle of reproductive assistance. Magnes. Res..

[115] Palmery M., Saraceno A., Vaiarelli A., Carlomagno G. (2013). Oral Contraceptives and Changes in Nutritional Requirements. Eur. Rev..

[116] YAKINCI G., PAÇ A., KüÇüKBAY F.Z., TAYFUN M., GüL A. (2011). Serum zinc, copper, and magnesium levels in obese children. Pediatr. Int..

[117] Hassan S.A., Ahmed I., Nasrullah A., Haq S., Ghazanfar H., Sheikh A.B., Zafar R., Askar G., Hamid Z., Khushdil A. (2017). Comparison of Serum Magnesium Levels in Overweight and Obese Children and Normal Weight Children. Cureus.

[118] Rodríguez-Morán M., Guerrero-Romero F. (2004). Elevated concentrations of TNF-alpha are related to low serum magnesium levels in obese subjects. Magnes. Res..

[119] Agarwal S., Reider C., Brooks J.R., Fulgoni V.L. (2015). Comparison of Prevalence of Inadequate Nutrient Intake Based on Body Weight Status of Adults in the United States: An Analysis of NHANES 2001–2008. J. Am. Coll. Nutr..

[120] Lowenstein F.W., Stanton M.F. (1986). Serum magnesium levels in the United States, 1971–1974. J. Am. Coll. Nutr..

[121] Kroll M.H., Elin R.J. (1985). Relationships between magnesium and protein concentrations in serum. Clin. Chem..

[122] Lim P., Jacob E., Dong S., Khoo O.T. (1969). Values for tissue magnesium as a guide in detecting magnesium deficiency. J. Clin. Pathol..

[123] Witkowski M., Hubert J., Mazur A. (2012). Methods of assessment of magnesium status in humans: A systematic review. Magnes. Res..

[124] Elin R.J. (2010). Assessment of magnesium status for diagnosis and therapy. Magnes. Res..

[125] Fairley J., Glassford N.J., Zhang L., Bellomo R. (2015). Magnesium status and magnesium therapy in critically ill patients: A systematic review. J. Crit. Care.

[126] Martínez A.C., Fernández-Lázaro D., Mielgo-Ayuso J., Calvo J.S., García A.C. (2017). Effect of magnesium supplementation on muscular damage markers in basketball players during a full season. Magnes. Res..

[127] Yeh D.D., Chokengarmwong N., Chang Y., Yu L., Arsenault C., Rudolf J., Lee-Lewandrowski E., Lewandrowski K. (2017). Total and ionized magnesium testing in the surgical intensive care unit—Opportunities for improved laboratory and pharmacy utilization. J. Crit. Care.

[128] Arnaud M.J. (2008). Update on the assessment of magnesium status. Br. J. Nutr..

[129] Basso L.E., Ubbink J.B., Delport R. (2000). Erythrocyte magnesium concentration as an index of magnesium status: A perspective from a magnesium supplementation study. Clin. Chim. Acta Int. J. Clin. Chem..

[130] Zhang X., Del Gobbo L.C., Hruby A., Rosanoff A., He K., Dai Q., Costello R.B., Zhang W., Song Y. (2016). The Circulating Concentration and 24-h Urine Excretion of Magnesium Dose- and Time-Dependently Respond to Oral Magnesium Supplementation in a Meta-Analysis of Randomized Controlled Trials. J. Nutr..

[131] Zemel P.C., Zemel M.B., Urberg M., Douglas F.L., Geiser R., Sowers J.R. (1990). Metabolic and hemodynamic effects of magnesium supplementation in patients with essential hypertension. Am. J. Clin. Nutr..

[132] Facchinetti F., Borella P., Sances G., Fioroni L., Nappi R.E., Genazzani A.R. (1991). Oral magnesium successfully relieves premenstrual mood changes. Obstet. Gynecol..

[133] Desbiens N.A., Marx J.J., Haas R.G., Reinhart R.A. (1992). Can the magnesium content of mononuclear blood cells be altered by oral magnesium supplementation?. Clin. Biochem..

[134] Ferrara L.A., Iannuzzi R., Castaldo A., Iannuzzi A., Russo D., Mancini M. (1992). Long-Term Magnesium Supplementation in Essential Hypertension. Cardiology.

[135] Bashir Y., Sneddon J.F., Staunton H.A., Haywood G.A., Simpson I.A., McKenna W.J., Camm A.J. (1993). Effects of long-term oral magnesium chloride replacement in congestive heart failure secondary to coronary artery disease. Am. J. Cardiol..

[136] Plum-Wirell M., Stegmayr B.G., Wester P.O. (1994). Nutritional magnesium supplementation does not change blood pressure nor serum or muscle potassium and magnesium in untreated hypertension. A double-blind crossover study. Magnes. Res..

[137] Witteman J.C., Grobbee D.E., Derkx F.H., Bouillon R., de Bruijn A.M., Bruijn D.A., Hofman A. (1994). Reduction of blood pressure with oral magnesium supplementation in women with mild to moderate hypertension. Am. J. Clin. Nutr..

[138] Eibl N.L., Kopp H.P., Nowak H.R., Schnack C.J., Hopmeier P.G., Schernthaner G. (1995). Hypomagnesemia in type II diabetes: Effect of a 3-month replacement therapy. Diabetes Care.

[139] Eriksson J., Kohvakka A. (1995). Magnesium and Ascorbic Acid Supplementation in Diabetes mellitus. Ann. Nutr. Metab..

[140] Itoh K., Kawasaka T., Nakamura M. (1997). The effects of high oral magnesium supplementation on blood pressure, serum lipids and related variables in apparently healthy Japanese subjects. Br. J. Nutr..

[141] Sanjuliani A.F., de Abreu Fagundes V.G., Francischetti E.A. (1996). Effects of magnesium on blood pressure and intracellular ion levels of Brazilian hypertensive patients. Int. J. Cardiol..

[142] Costello R.B., Moser-Veillon P.B., DiBianco R. (1997). Magnesium supplementation in patients with congestive heart failure. J. Am. Coll. Nutr..

[143] Sacks F.M., Willett W.C., Smith A., Brown L.E., Rosner B., Moore T.J. (1998). Effect on Blood Pressure of Potassium, Calcium, and Magnesium in Women with Low Habitual Intake. Hypertension.

[144] De Valk H.W., Verkaaik R., van Rijn H.J., Geerdink R.A., Struyvenberg A. (1998). Oral magnesium supplementation in insulin-requiring Type 2 diabetic patients. Diabet. Med. J. Br. Diabet. Assoc..

[145] De Lordes Lima M., Cruz T., Pousada J.C., Rodrigues L.E., Barbosa K., Canguçu V. (1998). The effect of magnesium supplementation in increasing doses on the control of type 2 diabetes. Diabetes Care.

[146] Walker A.F., De Souza M.C., Vickers M.F., Abeyasekera S., Collins M.L., Trinca L.A. (1998). Magnesium supplementation alleviates premenstrual symptoms of fluid retention. J. Womens Health.

[147] Weller E., Bachert P., Meinck H., Friedmann B., BÄrtsch P., MairbÄurl H. (1998). Lack of effect of oral Mg-supplementation on Mg in serum, blood cells, and calf muscle. Med. Sci. Sports Exerc..

[148] Hägg E., Carlberg B.C., Hillörn V.S., Villumsen J. (1999). Magnesium therapy in type 1 diabetes. A double blind study concerning the effects on kidney function and serum lipid levels. Magnes. Res..

[149] Wary C., Brillault-Salvat C., Bloch G., Leroy-Willig A., Roumenov D., Grognet J.M., Leclerc J.H., Carlier P.G. (1999). Effect of chronic magnesium supplementation on magnesium distribution in healthy volunteers evaluated by 31P-NMRS and ion selective electrodes. Br. J. Clin. Pharmacol..

[150] Zorbas Y.G., Kakurin V.J., Afonin V.B., Charapakhin K.P., Denogradov S.D. (1999). Magnesium supplements’ effect on magnesium balance in athletes during prolonged restriction of muscular activity. Kidney Blood Press. Res..

[151] Shechter M., Sharir M., Labrador M.J.P., Forrester J., Silver B., Merz C.N.B. (2000). Oral Magnesium Therapy Improves Endothelial Function in Patients With Coronary Artery Disease. Circulation.

[152] Walker A.F., De Souza M.C., Marakis G., Robinson P.A., Morris A.P., Bolland K.M. (2002). Unexpected benefit of sorbitol placebo in Mg intervention study of premenstrual symptoms: Implications for choice of placebo in RCTs. Med. Hypotheses.

[153] Mooren F.C., Golf S.W., Völker K. (2003). Effect of magnesium on granulocyte function and on the exercise induced inflammatory response. Magnes. Res..

[154] Rodríguez-Morán M., Guerrero-Romero F. (2003). Oral magnesium supplementation improves insulin sensitivity and metabolic control in type 2 diabetic subjects: A randomized double-blind controlled trial. Diabetes Care.

[155] Walker A.F., Marakis G., Christie S., Byng M. (2003). Mg citrate found more bioavailable than other Mg preparations in a randomised, double-blind study. Magnes. Res..

[156] Závaczki Z., Szöllõsi J., Kiss S.A., Koloszár S., Fejes I., Kovács L., Pál A. (2003). Magnesium-orotate supplementation for idiopathic infertile male patients: A randomized, placebo-controlled clinical pilot study. Magnes. Res..

[157] De Leeuw I., Engelen W., De Block C., Van Gaal L. (2004). Long term magnesium supplementation influences favourably the natural evolution of neuropathy in Mg-depleted type 1 diabetic patients (T1dm). Magnes. Res..

[158] Pokan R., Hofmann P., von Duvillard S.P., Smekal G., Wonisch M., Lettner K., Schmid P., Shechter M., Silver B., Bachl N. (2006). Oral magnesium therapy, exercise heart rate, exercise tolerance, and myocardial function in coronary artery disease patients. Br. J. Sports Med..

[159] Barragán-Rodríguez L., Rodríguez-Morán M., Guerrero-Romero F. (2008). Efficacy and safety of oral magnesium supplementation in the treatment of depression in the elderly with type 2 diabetes: A randomized, equivalent trial. Magnes. Res..

[160] Almoznino-Sarafian D., Sarafian G., Berman S., Shteinshnaider M., Tzur I., Cohen N., Gorelik O. (2009). Magnesium administration may improve heart rate variability in patients with heart failure. Nutr. Metab. Cardiovasc. Dis..

[161] Lee S., Park H.K., Son S.P., Lee C.W., Kim I.J., Kim H.J. (2009). Effects of oral magnesium supplementation on insulin sensitivity and blood pressure in normo-magnesemic nondiabetic overweight Korean adults. Nutr. Metab. Cardiovasc. Dis..

[162] Guerrero-Romero F., Rodríguez-Morán M. (2009). The effect of lowering blood pressure by magnesium supplementation in diabetic hypertensive adults with low serum magnesium levels: A randomized, double-blind, placebo-controlled clinical trial. J. Hum. Hypertens..

[163] Aydin H., Deyneli O., Yavuz D., Gözü H., Mutlu N., Kaygusuz I., Akalin S. (2010). Short-term oral magnesium supplementation suppresses bone turnover in postmenopausal osteoporotic women. Biol. Trace Elem. Res..

[164] Kazaks A.G., Uriu-Adams J.Y., Albertson T.E., Shenoy S.F., Stern J.S. (2010). Effect of oral magnesium supplementation on measures of airway resistance and subjective assessment of asthma control and quality of life in men and women with mild to moderate asthma: A randomized placebo controlled trial. J. Asthma Off. J. Assoc. Care Asthma.

[165] Nielsen F.H., Johnson L.K., Zeng H. (2010). Magnesium supplementation improves indicators of low magnesium status and inflammatory stress in adults older than 51 years with poor quality sleep. Magnes. Res..

[166] Zorbas Y.G., Kakuris K.K., Federenko Y.F., Deogenov V.A. (2010). Utilization of magnesium during hypokinesia and magnesium supplementation in healthy subjects. Nutrition.

[167] Chacko S.A., Sul J., Song Y., Li X., LeBlanc J., You Y., Butch A., Liu S. (2011). Magnesium supplementation, metabolic and inflammatory markers, and global genomic and proteomic profiling: A randomized, double-blind, controlled, crossover trial in overweight individuals. Am. J. Clin. Nutr..

[168] Guerrero-Romero F., Rodríguez-Morán M. (2011). Magnesium improves the beta-cell function to compensate variation of insulin sensitivity: Double-blind, randomized clinical trial. Eur. J. Clin. Investig..

[169] Tarighat Esfanjani A., Mahdavi R., Ebrahimi Mameghani M., Talebi M., Nikniaz Z., Safaiyan A. (2012). The effects of magnesium, L-carnitine, and concurrent magnesium-L-carnitine supplementation in migraine prophylaxis. Biol. Trace Elem. Res..

[170] Laecke S.V., Nagler E.V., Taes Y., Biesen W.V., Peeters P., Vanholder R. (2014). The effect of magnesium supplements on early post-transplantation glucose metabolism: A randomized controlled trial. Transpl. Int..

[171] Cosaro E., Bonafini S., Montagnana M., Danese E., Trettene M.S., Minuz P., Delva P., Fava C. (2014). Effects of magnesium supplements on blood pressure, endothelial function and metabolic parameters in healthy young men with a family history of metabolic syndrome. Nutr. Metab. Cardiovasc. Dis..

[172] Rodríguez-Moran M., Guerrero-Romero F. (2014). Oral Magnesium Supplementation Improves the Metabolic Profile of Metabolically Obese, Normal-weight Individuals: A Randomized Double-blind Placebo-controlled Trial. Arch. Med. Res..

[173] Setaro L., Santos-Silva P.R., Nakano E.Y., Sales C.H., Nunes N., Greve J.M., Colli C. (2014). Magnesium status and the physical performance of volleyball players: Effects of magnesium supplementation. J. Sports Sci..

[174] Navarrete-Cortes A., Ble-Castillo J.L., Guerrero-Romero F., Cordova-Uscanga R., Juárez-Rojop I.E., Aguilar-Mariscal H., Tovilla-Zarate C.A., del Rocio Lopez-Guevara M. (2014). No effect of magnesium supplementation on metabolic control and insulin sensitivity in type 2 diabetic patients with normomagnesemia. Magnes. Res..

[175] Guerrero-Romero F., Simental-Mendia L.E., Hernandez-Ronquillo G., Rodriguez-Moran M. (2015). Oral magnesium supplementation improves glycemic status in subjects with prediabetes and hypomagnesaemia: A double-blind placebo-controlled randomized trial. Diabetes Metab..

[176] Park H., Qin R., Smith T.J., Atherton P.J., Barton D.L., Sturtz K., Dakhil S.R., Anderson D.M., Flynn K., Puttabasavaiah S. (2015). NCCTG N10C2 (Alliance)—A Double-Blind, Placebo-Controlled Study of Magnesium Supplements to Reduce Menopausal Hot Flashes. Menopause NYN.

[177] Baker W.L., Kluger J., Coleman C.I., White C.M. (2015). Impact of Magnesium L-Lactate on Occurrence of Ventricular Arrhythmias in Patients with Implantable Cardioverter Defibrillators: A Randomized, Placebo-Controlled Trial. Open Cardiovasc. Med. J..

[178] Joris P.J., Plat J., Bakker S.J., Mensink R.P. (2016). Long-term magnesium supplementation improves arterial stiffness in overweight and obese adults: Results of a randomized, double-blind, placebo-controlled intervention trial. Am. J. Clin. Nutr..

[179] Terink R., Balvers M.G.J., Hopman M.T., Witkamp R.F., Mensink M., Gunnewiek J.M.T.K. (2016). Decrease in Ionized and Total Magnesium Blood Concentrations in Endurance Athletes Following an Exercise Bout Restores within Hours—Potential Consequences for Monitoring and Supplementation. Int. J. Sport Nutr. Exerc. Metab..

[180] Moradian S.T., Ghiasi M.S., Mohamadpour A., Siavash Y. (2017). Oral magnesium supplementation reduces the incidence of gastrointestinal complications following cardiac surgery: A randomized clinical trial. Magnes. Res..

[181] Rajizadeh A., Mozaffari-Khosravi H., Yassini-Ardakani M., Dehghani A. (2017). Effect of magnesium supplementation on depression status in depressed patients with magnesium deficiency: A randomized, double-blind, placebo-controlled trial. Nutrition.

[182] Cunha A.R., D’El-Rei J., Medeiros F., Umbelino B., Oigman W., Touyz R.M., Neves M.F. (2017). Oral magnesium supplementation improves endothelial function and attenuates subclinical atherosclerosis in thiazide-treated hypertensive women. J. Hypertens..

[183] Bressendorff I., Hansen D., Schou M., Kragelund C., Brandi L. (2017). The effect of magnesium supplementation on vascular calcification in chronic kidney disease—A randomised clinical trial (MAGiCAL-CKD): Essential study design and rationale. BMJ Open.

[184] Bressendorff I., Hansen D., Schou M., Silver B., Pasch A., Bouchelouche P., Pedersen L., Rasmussen L.M., Brandi L. (2017). Oral Magnesium Supplementation in Chronic Kidney Disease Stages 3 and 4: Efficacy, Safety, and Effect on Serum Calcification Propensity—A Prospective Randomized Double-Blinded Placebo-Controlled Clinical Trial. Kidney Int. Rep..

[185] Toprak O., Kurt H., Sarı Y., Şarkış C., Us H., Kırık A. (2017). Magnesium Replacement Improves the Metabolic Profile in Obese and Pre-Diabetic Patients with Mild-to-Moderate Chronic Kidney Disease: A 3-Month, Randomised, Double-Blind, Placebo-Controlled Study. Kidney Blood Press. Res..

[186] Joosten M.M., Gansevoort R.T., Mukamal K.J., van der Harst P., Geleijnse J.M., Feskens E.J., Navis G., Bakker S.J. (2013). Urinary and plasma magnesium and risk of ischemic heart disease. Am. J. Clin. Nutr..

[187] Djurhuus M.S., Gram J., Petersen P.H., Klitgaard N.A.H., Bollerslev J., Beck-Nielsen H. (1995). Biological variation of serum and urinary magnesium in apparently healthy males. Scand. J. Clin. Lab. Investig..

[188] Tahiri M., Tressol J.C., Arnaud J., Bornet F., Bouteloup-Demange C., Feillet-Coudray C., Ducros V., Pépin D., Brouns F., Roussel A.M. (2001). Five-Week Intake of Short-Chain Fructo-Oligosaccharides Increases Intestinal Absorption and Status of Magnesium in Postmenopausal Women. J. Bone Miner. Res..

[189] Saur P.M., Zielmann S., Roth A.T., Frank L., Warneke G., Radke A., Ensink F.B., Kettler D. (1996). Diagnosis of magnesium deficiency in intensive care patients. Anasthesiologie Intensivmedizin Notfallmedizin Schmerztherapie AINS.

[190] Hébert P., Mehta N., Wang J., Hindmarsh T., Jones G., Cardinal P. (1997). Functional magnesium deficiency in critically ill patients identified using a magnesium-loading test. Crit. Care Med..

[191] Saur P., Niedmann P.D., Brunner E., Kettler D. (2005). Do intracellular, extracellular or urinary magnesium concentrations predict renal retention of magnesium in critically ill patients?. Eur. J. Anaesthesiol..

[192] Wälti M.K., Walczyk T., Zimmermann M.B., Fortunato G., Weber M., Spinas G.A., Hurrell R.F. (2006). Urinary excretion of an intravenous ^26^Mg dose as an indicator of marginal magnesium deficiency in adults. Eur. J. Clin. Nutr..

[193] Martin B.J. (1990). The magnesium load test: Experience in elderly subjects. Aging Clin. Exp. Res..

[194] Haigney M.C.P., Silver B., Tanglao E., Silverman H.S., Hill J.D., Shapiro E., Gerstenblith G., Schulman S.P. (1995). Noninvasive Measurement of Tissue Magnesium and Correlation with Cardiac Levels. Circulation.

[195] Coudray C., Rambeau M., Feillet-Coudray C., Tressol J.C., Demigne C., Gueux E., Mazur A., Rayssiguier Y. (2005). Dietary inulin intake and age can significantly affect intestinal absorption of calcium and magnesium in rats: A stable isotope approach. Nutr. J..

[196] Sojka J., Wastney M., Abrams S., Lewis S.F., Martin B., Weaver C., Peacock M. (1997). Magnesium kinetics in adolescent girls determined using stable isotopes: Effects of high and low calcium intake. Am. J. Physiol. Regul. Integr. Comp. Physiol..

[197] Abrams S.A. (2009). 16—Using stable isotopes to determine mineral bioavailability of functional foods. Designing Functional Foods.

[198] Hachey D.L., Wong W.W., Boutton T.W., Klein P.D. (1988). Stable isotopes in the study of human nutrition. Int. J. Radiat. Appl. Instrum..

[199] Hansen K.E., Nabak A.C., Johnson R.E., Marvdashti S., Keuler N.S., Shafer M.M., Abrams S.A. (2014). Isotope Concentrations from 24-h Urine and 3-h Serum Samples Can Be Used to Measure Intestinal Magnesium Absorption in Postmenopausal Women. J. Nutr..

[200] Draxler J., Martinelli E., Weinberg A.M., Zitek A., Irrgeher J., Meischel M., Stanzl-Tschegg S.E., Mingler B., Prohaska T. (2017). The potential of isotopically enriched magnesium to study bone implant degradation in vivo. Acta Biomater..

[201] Avioli L.V., Berman M. (1966). Mg28 kinetics in man. J. Appl. Physiol..

[202] Danielson B.G., Johansson G., Ljunghall S. (1979). Magnesium metabolism in healthy subjects. Scand. J. Urol. Nephrol. Suppl..

[203] Watson W.S., Hilditch T.E., Horton P.W., Davies D.L., Lindsay R. (1979). Magnesium metabolism in blood and the whole body in man using 28magnesium. Metabolism.

[204] Costello R.B., Nielsen F. (2017). Interpreting magnesium status to enhance clinical care: Key indicators. Curr. Opin. Nutr. Metab. Care.

[205] Bilbey D.L., Prabhakaran V.M. (1996). Muscle cramps and magnesium deficiency: Case reports. Can. Fam. Physician.

[206] Abbasi B., Kimiagar M., Sadeghniiat K., Shirazi M.M., Hedayati M., Rashidkhani B. (2012). The effect of magnesium supplementation on primary insomnia in elderly: A double-blind placebo-controlled clinical trial. J. Res. Med. Sci. Off. J. Isfahan Univ. Med. Sci..

[207] Romano T.J., Stiller J.W. (1994). Magnesium Deficiency in Fibromyalgia Syndrome. J. Nutr. Med..

[208] Moorkens G., Manuel B.Y.K., Vertommen J., Meludu S., Noe M., De I.L. (1997). Magnesium deficit in a sample of the Belgian population presenting with chronic fatigue. Magnes. Res..

[209] Kumeda Y., Inaba M. (2005). Metabolic syndrome and magnesium. Clin. Calcium.

